# Recent advances on uric acid transporters

**DOI:** 10.18632/oncotarget.20135

**Published:** 2017-08-10

**Authors:** Liuqing Xu, Yingfeng Shi, Shougang Zhuang, Na Liu

**Affiliations:** ^1^ Department of Nephrology, Shanghai East Hospital, Tongji University School of Medicine, Shanghai 200120, China; ^2^ Department of Medicine, Rhode Island Hospital and Brown University School of Medicine, Providence, RI 02903, USA

**Keywords:** hyperuricemia, uric acid transporter protein, structure and function

## Abstract

Uric acid is the product of purine metabolism and its increased levels result in hyperuricemia. A number of epidemiological reports link hyperuricemia with multiple disorders, such as kidney diseases, cardiovascular diseases and diabetes. Recent studies also showed that expression and functional changes of urate transporters are associated with hyperuricemia. Uric acid transporters are divided into two categories: urate reabsorption transporters, including urate anion transporter 1 (URAT1), organic anion transporter 4 (OAT4) and glucose transporter 9 (GLUT9), and urate excretion transporetrs, including OAT1, OAT3, urate transporter (UAT), multidrug resistance protein 4 (MRP4/ABCC4), ABCG-2 and sodium-dependent phosphate transport protein. In the kidney, uric acid transporters decrease the reabsorption of urate and increase its secretion. These transporters’ dysfunction would lead to hyperuricemia. As the function of urate transporters is important to control the level of serum uric acid, studies on the functional role of uric acid transporter may provide a new strategy to treat hyperuricemia associated diseases, such as gout, chronic kidney disease, hyperlipidemia, hypertension, coronary heart disease, diabetes and other disorders. This review article summarizes the physiology of urate reabsorption and excretion transporters and highlights the recent advances on their roles in hyperuricemia and various diseases.

## INTRODUCTION

In recent years, along with the improvement of living standard and the alteration in diet, elevation of food intake rich in protein and purine results in increased incidence of hyperuricemia [1]. Hyperuricemia, defined as uric acid level over 7.0 mg/dL in men and 6.0 mg/dL in women [2], is critically associated with gout. Owing to lack of uricase, uric acid is the final metabolic product of purine metabolism in human [3]. In other mammals, uric acid is degraded into allantoin through uricase in the liver. Thus, in the process of human evolution, due to the antioxidant effect of urate, a higher level of serum urate might be a selection advantage [4]. However, the high level of urate is harmful to human body [5–13]. As the blood level of uric acid is tightly regulated by its reabsorption and excretion in the kidney, we here review recent advances on the role of urate reabsorption transporters and urate excretion transporters in the kidney and discuss the link between hyperuricemia and various diseases, in particular, renal diseases.

## CLASSIFICATION OF URIC ACID TRANSPORTERS

Excretion of uric acid in the kidney is carried out with the assistance of uric acid transporters, and the transporters are divided into two categories: urate reabsorption transporters and urate excretion transporters [14].

Urate reabsorption transporters have three members, urate anion transporter 1 (URAT1), organic anion transporter 4 (OAT4) and glucose transporter 9 (GLUT9). URAT1 has been identified as a new member of the organic anion transporter family [15, 16]. OAT4 belongs to solute carrier family. As a member of GLUT family, GLUT9 has a high capacity for transporting urate in humans and increases the speed of uric acid reabsorption by glucose transport [17].

Urate excretion transporters also include two categories: the uptake of uric acid transporters and the excretion of uric acid transporters. To date, more than ten OAT species have been identified [18]. The uptake of uric acid from blood to intracellular tubular cells is mainly assisted by OAT1 and OAT3. Excretion of uric acid from tubular cells to their lumens is carried out by four transporters, they are urate transporter (UAT), multidrug resistance protein 4 (MRP4/ABCC4), ABCG-2 and sodium-dependent phosphate transport protein. OAT3 is found in some organs like kidney, liver, brain and eyes, while OAT1 is mostly located in the kidney. OAT1 and OAT3 start to work depending on the concentration gradient of oxygen glutaric acid between intracellular and interstitial tissue. UAT is a specific excretion of uric acid transporter, which plays an important role in regulating homeostasis of body uric acid. MRP4 is a transporter encoded by the ABCG4 gene, which is included in the ATP-binding cassette family. ATP depletion can provide energy for its transporting function. This feature is distinct from the concentration gradient-dependent OAT1 and OAT3. Like MRP4, ABCG2 is also an ATP-binding cassette half-transport protein with its gene being located on 4q22 [14]. (Table 1).

**Table 1:** Classification of urate transporters

Classification of urate transporters			The main distribution
Urate reabsorption transporter	Urate anion transporter 1(URAT1)		Located in the lumen side of the proximal tubular epithelial cells membrane
	Urate anion transporter 1(URAT1)		Expressed in both lumen and basolateral side of the proximal tubular epithelial cells membrane
Urate excretion transporter	Tubular cell uptake of uric acid transporters	Organic anion transporter 1(OAT1)	Expressed inbasolateral side of the proximal tubular epithelial cells membrane
		Organic anion transporter 3(OAT3)	Expressed inbasolateral side of the proximal tubular epithelial cells membrane
	Tubular cell secretion of uric acid transporters	Urate transporter	Expressed inbasolateral side of the proximal tubular epithelial cells membrane
		Multidrug resistance protein 4(MRP4)	Located in the tubular epithrlial cells brush border
		ABCG-2	Located in both apical and basolateral side of the proximal tubular epithelial cells membrane
		Glucose transporter 9(GLUT9)	Located in both apical and basolateral side of the proximal tubular epithelial cells membrane

## THE STRUCTURE AND FUNCTION OF URATE TRANSPORTERS

Uric acid crosses through the glomerulus freely, then most is reabsorbed by proximal tubular urate transporters and only a small portion of it is secreted back into the filtrate via the late proximal tubule [19]. The urate transport models found in the proximal tubule suggest that the process of initial uptake of uric acid by URAT1 is associated with organic acid transporters. GLUT9 has two different isoforms, one is GLUT9-L, and another is GLUT9-S. Urate getting out the basement membrane of the proximal tubules is regulated by GLUT9-L, while urate getting in and out of the apical membrane is controlled by GLUT9-S. In the distal tubule, some transporters such as ABCG2, NPT1 and NPT4 mediate uric acid secretion [20].

### Urate reabsorption transporters

#### Urate anion transporter 1

URAT1 (SLC22A12 gene), the major urate reabsorption transporter, was first identified in Xenopus oocytes. The researchers have discussed organic anion transporter-like (OAT-like) molecules in two respects, one is a gene database and the other is their expression/function [16]. URAT1 is a protein with 12-transmembrane domains, including 555 amino acid residues [16, 21]. It is found in the apical membrane of proximal tubule epithelial cells and interchanges uric acid in the proximal tubular lumen with Cl^−^ or organic anions in the epithelial cells. URAT1, specifically expressed at the side of the proximal tubular luminal membrane, is the key to maintain serum uric acid level by reabsorbing urate from the proximal tubule to the epithelia cells. Some uric acid excretion agents such as probenecid, sulfinpyrazone and benzbromarone promote the excretion of uric acid by combining with URAT1 to interrupt the reabsorption of uric acid [22]. Moreover, the angiotensin II receptor blocker losartan also increases the excretion of uric acid and lowers blood uric acid level by combining with URAT1 [14].

#### Organic anion transporter 4

As a multiple specific anion transporter, organic anion transporter 4 (encoded by the SLC22A11 gene) is located in the apical membrane of epithelial cells [23, 24]. It plays a role in urate reabsorption in lumen by an intracellular dicarboxylate gradient.

#### Glucose transporter 9

Glucose transporter 9 (SLC2A9), as a member of the glucose transporter (GLUT) family [25], has the structure of a type II Glut isoform, with 12-transmembrane domains. Between the first and second transmembrane domains, there is a large extracellular loop, and both amino and carboxyterminal end on the cytoplasmic side [26]. In fact, GLUT9 was originally identified as a glucose and/or fructose transporter, rather than a urate transporter. Like other members of the GLUT family, GLUT9 could also be inhibited by cytochalasin B. Studies from Caulfield’s group suggested that GLUT9 was a high-capacity urate transporter protein, which could accelerate reabsorption of urate acid by transporting glucose [17]. GLUT9 has two different isoforms, one is SLC2A9-L (540 amino acids) and the other is SLC2A9-S (512 amino acids). Toru Kimura et al. found that the locations of these two isoforms are different, SLC2A9-L located at the basolateral membrane of proximal tubules, while SLC2A9-S located at the apical membrane of collecting ducts [27]. As further studies found that some kinetic properties of GLUT9 isoforms could not be distinguished from each other. Besides, as GLUT9 could be activated by membrane depolarization, it is more like an electrogenic exchanger than a urate-anion exchanger. The mechanism by urate exits via electrogenic GLUT9 from the tubule cells might due to the negative membrane potential of proximal tubule cells [27, 28].

A genome-wide prospective study declared that SLC2A9 was a major gene determining the level of serum uric acid, which could explain about 3.5% changes of serum uric acid levels [29]. A recent study demonstrated that SLC2A9 single-nucleotide polymorphisms (SNPs) were associated with the level of serum uric acid [30]. Another study showed that hypouricemia induced by the SLC2A9 mutation, might associate with decreased reabsorption function, due to the loss-of-function of GLUT9 [31]. In addition, it was found that knocking out the systemic GLUT9 in mice resulted in blockage of uric acid reabsorption [32, 33].

### Urate excretion transporters

#### Tubular cell uptake of uric acid transporters

The organic anion and urate transporters OAT1 (SLC22A6) and OAT3 (SLC22A8) are expressed at the basolateral side of the same cells that express OAT4 [24, 34, 35] and have a great influence on urate excretion as urate/dicarboxylate exchangers [16, 36, 37]. However, in the rat kidney, OAT3 is also identified both in the proximal tubule and the collecting duct [38]. Cha et al. [39] demonstrated that OAT3 was a 12-transmembrane domain-containing protein. Alcohol is metabolized to lactic acid *in vivo*, the latter not only increases organic anion gradient in the renal epithelial cells, promotes the uric acid reabsorption, but competes for OAT1 and OAT3 with uric acid. Studies about gene knockout mice have shown that lack of OAT1 or OAT3 has a weak effect on decreasing uricosuria, advising that the primary function of them is to control urate secretion [40]. OAT1 and OAT3, on the basolateral membrane of epithelial cells, transport uric acid from the renal interstitial into tubular epithelial cells. The transfer power might come from the oxygen glutarate concentration gradient in intracellular and interstitial. The current studies illustrated that in hyperuricemia, expression of OAT1 and OAT3 was decreased [7].

#### Tubular secretion of uric acid transporters

#### Urate transporter

Urate transporter, first identified in the brush border of rat renal tubular epithelial cells, is a specific secretion of uric acid transporter protein. UAT is a galectin, a protein with two β-galactoside binding domains that bind lactose. With lactose, UAT open probability can increase markedly. Leal et al. found that UAT is a 322-amine acid protein, expressing in planar lipid bilayers as urate channel with highly selective and voltage sensitivity [41], but only functions in the channel′s extracellular side [42]. Thus it is suggested that UAT is not only a transporter associated with urate efflux from systemic cells but also an electrogenic urate transporter. Furthermore, recent studies suggested that among numerous species, UAT is an integral plasma membrane protein which has an intracellular termini in epithelial cells, regardless on whether the cells are renal or nonrenal [43].

Leal et al. [41] also showed that the two β-galactoside binding sites were outside of the cells, and could change the function of UAT after combinding with some specific substrates. Uricase inhibitor, such as Oxonate, PZA and adenosine, could inhibit the activity of recombined UAT channel.

#### Multidrug resistance protein 4

The multidrug resistance protein (MRP4/ABCC4) plays a role in urate secretion by the proximal tubule [44]. Human MRP4, locating in HEK293 cells [45], has an effect on urate excretion by promoting ATP-dependent urate extrusion from the cells into the tubule lumen thus contributing to urate excretion [46, 47]. In addition, Hoque et al. [48] studies proposed that some organic anions like cAMP, cGMP, and methotrexate could also be secreted into the tubule lumen by MRP4. Unlike OATs, MRP4 provides energy through the consumption of ATP. Benzbromarone and probenecid inhibit the function of human MRP4, due to an abnormal activation by allopurinol, the xanthine oxidase inhibitor, and its active metabolite, oxypurinol [45].

#### ABCG2

**Figure 1:**
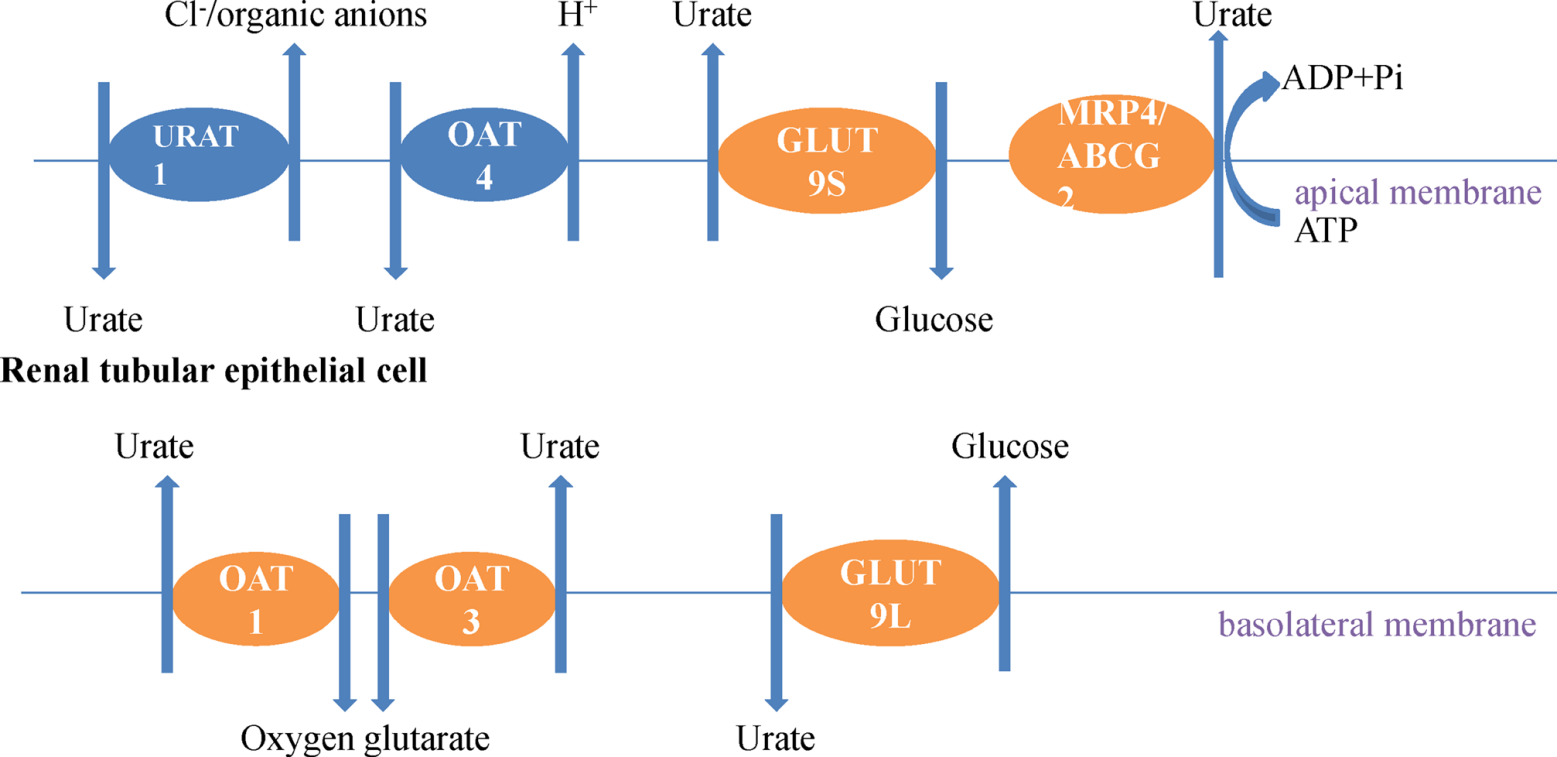
Uric acid transports in kidney epithelial cells The urate reabsorption pathway involves the apical exchanger proteins URAT1 and OAT4; intracellular urate is excreted through basolateral GLUT9L. Some uric acid excretion agents such as probenecid, sulfinpyrazone, benzbromarone and NSAIDs promote the excretion uric acid by combining with URAT1 to interrupt the reabsorption of uric acid. The excretion pathway critically involves the basolateral exchanger proteins OAT1 and OAT3, and the apical ATP-binding cassette proteins MRP4 and ABCG2.

ABCG2, another ATP-binding cassette of half-transporter protein, is identified in the researches of hyperuricemia and gout in gene level. ABCG2 locates in a small region of chromosome 4 [49]. Dehghan et al. [29] established the first nucleotide polymorphisms (SNPs), which associated with higher concentration of serum uric acid, by using a genetic screening tool, the genome-wide association study (GWAS). Functional studies [50] illustrated that ABCG2, expressing in the apical membrane of proximal collecting duct cells [51], decreased serum uric acid levels as a urate transporter [52]. However, recent studies substantiated that the mutations of ABCG2 could cause hyperuricemia [35, 50]. A general nonsynonymous SNP in ABCG2 Q141k, had a negative effect on ABCG2 function [45]. Following studies in Xenopus oocytes and mammalian cells [53, 54] indicated that there exists a defect, temperature-dependent expression, in ABCG2 Q141k, associated with unsteadiness of the nucleotide-binding domain [54]. Furthermore, only in the form of homodimer, ABCG2 has the activity of transporting uric acid (Figure 1).

## THE IMPACTS OF HYPERURICEMIA ON BODY

### Hyperuricemia leads to renal diseases

Recent studies have reported that high level of uric acid plays a direct detrimental role both in chronic kidney diseases and acute kidney injury. Thus hyperuricemia has been considered as an independent risk factor in renal disease progression [55]. Elevation of the serum uric acid level induces oxidative stress and endothelial dysfunction, resulting in the development of both systemic and glomerular hypertension combined with elevated renal vascular resistance and reduction of renal blood flow [56–58]. Hyperuricemia is also able to induce an epithelial-to-mesenchymal transition, which has direct effects on the tubular epithelial cell injury [59]. Lack of functional URAT1 transporters has some deleterious effects, such as lower blood levels of urate and higher urinary levels of urate, associated with the crystal formation within the renal tubules. Further studies have shown that hyperuricemia can accelerate glomerular hypertension and the vascular lesions, resulting in worsening proteinuria and renal dysfunction [60]. Uric acid has also been shown to activate the cytoplasmic phospholipase A2 and inflammatory transcription factor nuclear factor-κB (NF-κB), leading to the inhibition of proximal tubular cellular proliferation *in vitro* [61]. In addition, there are also reports showing that elevated serum uric acid stimulated sequelae cytokine production, such as tumor necrosis factor α [62], and the local expression of chemokines, such as monocyte chemotactic protein 1 in the kidney [63] and cyclooxygenase 2 (COX-2) in the blood vessels [64].

Hyperuricemia is very common in chronic kidney disease (CKD) owing to reduction of uric acid clearance [65, 66]. Although it has been not confirmed that uric acid has an effect on the progression of CKD, increasing evidence indicates that sustained uric acid is a risk factor that causes or exacerbates kidney fibrosis in progressive CKD [7]. Up-regulating the uric acid level in rats induces glomerular hypertension and renal diseases as noted by the development of arteriolosclerosis, glomerular injury and tubulointerstitial fibrosis [67], such effects have been shown to reduce glomerular filtration rate (GFR) [60]. A pilot study suggested that decreasing serum uric acid levels can slow the progression of renal disease in patients with chronic kidney disease [67]. As renal elimination has a primary effect on maintaining the level of serum uric acid, it is important to decrease uric acid reabsorption or increase uric acid excretion. It has been demonstrated that the novel Human uric acid transporter 1 (hURAT1) inhibitors could inhibit uric acid re-absorption significantly *in vivo* and *in vitro* [68, 69]. The results provide a new strategy in decreasing serum uric acid level through inhibiting uric acid reabsorption.

### The relationship between hyperuricemia and hyperlipidemia

Recently, Barlow et al. [70] revealed that there was a close correlation between hyperlipidemia and hyperuricemia. More than 80% patients with hyperlipidemia suffer from hyperuricemia while about 50% to 75% patients with hyperuricemia have hyperlipidemia. Numerous studies have indicated that elevated levels of serum uric acid up regulates the concentration of some lipid profiles, such as total cholesterol (TC), triglyceride (TG), very low-density lipoprotein cholesterol (VLDL), low-density lipoprotein cholesterol (LDL-C), while down regulate the concertration of high-density lipoprotein cholesterol (HDL-C) [71–73]. The major mechanism of hyperuricemia associated hyperlipidemia is an abnormal lipids metabolism. A large amount of VLDL in the blood decreases the expression of OAT1 in the HK2 cells, which results in reduced secretion of uric acid. Furthermore, hyperuricemia has a positive relationship with TG [74]. Takahashi et al. [75] showed that a higher level of TG might be associated with increased concentrations of LDL-C in hyperuricemic patients. Hyperuricemia closely relates to obesity due to increased energy intake and purine synthesis that results in producing more uric acid. When the obese patients are in a state of hungry and tired, the body will utilize the accumulated fat to produce heat to provide the body’s needs. However, at the same time, ketone bodies produced by decomposition of lipid would interfere with the excretion of serum uric acid, increasing the level of uric acid indirectly. In addition, the free fatty acids induced by metabolic syndrome into by-products, would decrease uric acid excretion, and increase serum uric acid. Furthermore, hyperlipidemia could induce glomerulosclerosis, and accelerate the progression of CKD. Meanwhile, hyperlipoidemia due to elevation of serum uric acid promotes the oxidation of low-density lipoprotein (LDL) cholesterol and lipid peroxidation.

### The relationship between hyperuricemia and atherosclerosis

Atherosclerosis is one of the most common cardiovascular diseases. It can trigger renal artery stenosis, decrease the blood flow speed of renal vessels, and also tightly correlate with acute or chronic kidney diseases. Patterson et al. demonstrated that uric acid performed as an antioxidant in the presence of native LDL, while as a pro-oxidant in response to mildly oxidized LDL when the oxidation has occurred [8]. Recent study shows that oxidized low-density lipoprotein had a pivotal effect on the formation of atheromatous plaques. Although LDL (ox-LDL) is extremely fundamental to the development of atherosclerosis through damaging endothelial cells and vascular smooth muscle cells, uric acid can induce the up-regulation of C-reactive protein in both vascular smooth muscle cells and endothelial cells, which add to the pro-atherogenic properties of soluble uric acid [76].

### The relationship between hyperuricemia and coronary heart disease

As one of major complication of CKD, coronary heart disease (CHD) is a disorder lacking of blood supplement to the heart muscle [77, 78]. Atherosclerotic lesions and vessel narrowing in the coronary arteries decrease blood supply. Hyperuricemia, in association with higher mortality rates in women with CKD, has been shown to act as a stimulating factor to individuals at risk of CHD related-events [79]. This was clearly suggested by a meta-analysis that investigated the connection between hyperuricemia and CHD, in which there is an increased CHD risk ratio of 1.12 in men and 1.22 in women with hyperuricemia [76].

Recently, Yongfeng et al. proceeded an investigation in middle aged and elderly Chinese, they wanted to identify the association between serum uric levels and cardiovascular disease. In the end, their data showed a slight positive relationship between serum uric acid level and heart failure or CHD in the patients who were adjusted for multi-variables [80]. Bjornstad et al. [81] demonstrated that serum uric acid was an independent predictor of vascular complications in type I diabetes.

Although several studies demonstrated that there is a connection between hyperuricemia and CHD, the functional role of serum uric acid in cardiovascular disease remains obscure [82]. Some studies demonstrated that inflammation might be a mechanism for hyperuricemia in promoting cardiovascular disease [83]. Reactive oxygen species (ROS), a production after xanthine oxidase (XO) activated, is acknowledged as one of the primary causes inducing endothelial dysfunction and vascular inflammation [84]. Uric acid has a negative affection on vascular function by inducing oxidant and decreasing nitric oxide bioavailability, and as a result function disorders occur in the endothelium. This property of uric acid declares a link between hyperuricemia and coronary heart disease [85].

### The relationship between hyperuricemia and diabetes

A series of longitudinal studies have declared that there is a connection between high levels of serum uric acid and high-risk of diabetic nephropathy complications among type I diabetes [86–88]. Thus, decreasing uric acid level should be tested as a novel intervention for diabetic nephropathy. Using allopurinol, organized by the Preventing Early Renal Function Loss (PERL) Consortium, is one of the tests. Since a high level of serum uric acid was observed in the most of patients with the type I diabetes, allopurinol might be used as a renoprotective medicine in diabetic patients [89].

Several studies have also demonstrated that higher serum urate acid concentration is linked to type II diabetes and the metabolic syndrome [64, 90]. Vuarinen-Markkola et al. showed that concentration of serum uric acid is related to insulin sensitivity and blood triglycerides. Urate crystals deposited in islet β cells of pancreas causes islet dysfunction, and consequently induces diabetes. In patients with diabetes, hyperuricemia, as a character of hyperinsulinemia or insulin resistance [29], is related with glycosuria [65], declined metabolic control, hyperfiltration, and a late commencement of disease [66]. Fructose, an endogenous production, increased by the polyol pathway activation. Meanwhile, it is an integral part of added sugars and the ability to engender urate is different with other sugars as a catabolite [67].

On the other hand, elevated blood glucose level can decrease the ability of urate transporter GLUT9, further worsening the hyperuricemia, and resulting in renal disfunction. Another mechanism by which hyperuricemia promotes the development of diabetes may be through inhibition of nitric oxide synthase [91, 92]. This is supported by the observation that the nitric oxide synthase knockout mice showed special characteristics of metabolic syndrome including hypertension, hypercholesterolemia, hypertriglyceridemia, and increased insulin resistance [93].

## CONCLUSIONS

Recently, the number of studies related to transport and mechanisms of uric acid is increasing. However, there are some difficulties in the study of hyperuricemia, the major one is the difference of uric acid metabolism between human and experimental animals, such as rats. The level of uric acid in rats is lower than that in human because uricase is present in rats but not in human and uricase can degrade uric acid into allantoin [94]. There are also some limitations of the current animal model of hyperuricemia. The first is the selection of experimental animals. Rat and mice are common experimental animals due to lower price. But the end products of uric acid in them are allantoin, which is different from human. The second one is the method for establishing hyperuricemia animal model. There are four common methods reported in the literature, including using the inhibitor of urate oxidase [95–97], feeding purine-rich foods [98], administration of a mixture with adenine and potassium oxonate [99–102] and disrupting the Uox gene which leads to uricase deficient in mice [103–105]. However, too much purine *in vivo* converts into dihydroxy adenine, deposits in the kidney and then causes kidney injury. Moreover, the effect of using the inhibitor of urate oxidase along on serum uric acid is mild [92].Uric acid transporter has been focused as its function of transporting some medicines, associated with the distribution and secretion of those medicines [106]. In the kidney, the filtrated urate can be reabsorbed in the proximal tubules in humans, while is secreted into the tubule fluid in other species. Uric acid transporters that reside in apical or basolateral membrane regulate the reabsorption and secretion of urate. Under physiological conditions, these transporters function coordinately keep the balance of uric acid *in vivo*. However, when these transporters lose their function, excessive uric acid will be accumulated in the body, leading to hyperuricemia. Since the function of urate transports is essential for determining serum uric acid concentrations, further studies on the functional role of uric acid transporter will provide a novel strategy to treat hyperuricemia associated diseases, such as gout, chronic kidney disease, hyperlipidemia, hypertension, coronary heart disease, diabetes and other disorders [53].
